# Autophagy Process in Trophoblast Cells Invasion and Differentiation: Similitude and Differences With Cancer Cells

**DOI:** 10.3389/fonc.2021.637594

**Published:** 2021-04-15

**Authors:** Lorena Carvajal, Jaime Gutiérrez, Eugenia Morselli, Andrea Leiva

**Affiliations:** ^1^ Department of Physiology, Faculty of Biological Sciences, Pontificia Universidad Católica de Chile, Santiago, Chile; ^2^ School of Medical Technology, Health Sciences Faculty, Universidad San Sebastian, Santiago, Chile; ^3^ Autophagy Research Center, Santiago, Chile

**Keywords:** autophagy, placentation, trophoblast and cancer cells, cellular proliferation, migration and invasion, vasculogenic capacity, vascular remodeling, immune evasion

## Abstract

Early human placental development begins with blastocyst implantation, then the trophoblast differentiates and originates the cells required for a proper fetal nutrition and placental implantation. Among them, extravillous trophoblast corresponds to a non-proliferating trophoblast highly invasive that allows the vascular remodeling which is essential for appropriate placental perfusion and to maintain the adequate fetal growth. This process involves different placental cell types as well as molecules that allow cell growth, cellular adhesion, tissular remodeling, and immune tolerance. Remarkably, some of the cellular processes required for proper placentation are common between placental and cancer cells to finally support tumor growth. Indeed, as in placentation trophoblasts invade and migrate, cancer cells invade and migrate to promote tumor metastasis. However, while these processes respond to a controlled program in trophoblasts, in cancer cells this regulation is lost. Interestingly, it has been shown that autophagy, a process responsible for the degradation of damaged proteins and organelles to maintain cellular homeostasis, is required for invasion of trophoblast cells and for vascular remodeling during placentation. In cancer cells, autophagy has a dual role, as it has been shown both as tumor promoter and inhibitor, depending on the stage and tumor considered. In this review, we summarized the similarities and differences between trophoblast cell invasion and cancer cell metastasis specifically evaluating the role of autophagy in both processes.

## Introduction

The placentation is a complex process that involves different stages, which quickly and efficiently leads to the development of the placenta, a temporary organ. The placenta is developed through regulated and dynamic cellular processes that include embryo pre-implantation and implantation, decidua formation, trophoblast proliferation, trophoblast differentiation into the invasive phenotype, and vascular remodeling (1). Interestingly, during placentation, the ability of trophoblast cells to proliferate, invade, and evade the immune system, resemble those induced by cancer cells during tumor growth (2). Indeed, the processes of proliferation, migration, and invasion in cancer cells and trophoblast derived cells share different molecules such as growth factors, cell adhesion molecules, surface receptors, matrix-digesting enzymes, and enzymes inhibitors, proto-oncogenes, hormones, and peptides, among others (3). These molecules regulate different processes that are highly controlled in trophoblasts, with trophoblast-derived cells following an organized pattern without metastasizing to new tissues, while the same pathways are dysregulated in cancer, driving metastasis (4).

In addition to sharing proliferative and invasive features, trophoblasts and cancer cells, actively modulate the host immune response to develop and sustain nutrient supply (5). Interestingly, it has been described that activation of autophagy occurs in both processes, regulating placental and cancer development (6, 7). However, how autophagy modulation affects trophoblast function is not entirely known (8). Consistently, the role of autophagy in cancer development is still a matter of study due to its dual role in tumor onset and progression (9, 10). Indeed, the role of autophagy in tumor development is controversial and dependent on tumor stage and type. It has been suggested that autophagy could promote aggressive characteristics of cancer cells, such as increased cellular invasion (11, 12), but also be a barrier to cancer proliferation (13–15). Additionally, autophagy also provides the microenvironment for placentation and cancer growth. This review will summarize the parallels between trophoblast-derived cells in placentation and cancer cells in tumor growth and metastasis with a final focus on the role of autophagy in both processes.

## Development of the Human Placenta

The placenta is a temporary organ that maintains and protects the fetus during pregnancy controlling the maternal-fetal exchange of nutrients, gases, and metabolic waste. Human pregnancy begins with the physiological preparation of the endometrium modulated by hormones such as progesterone and estrogen, which regulate growth factors, cytokines, and adhesion molecules that allow the blastocyst’s implantation (16).

**Figure 1:**
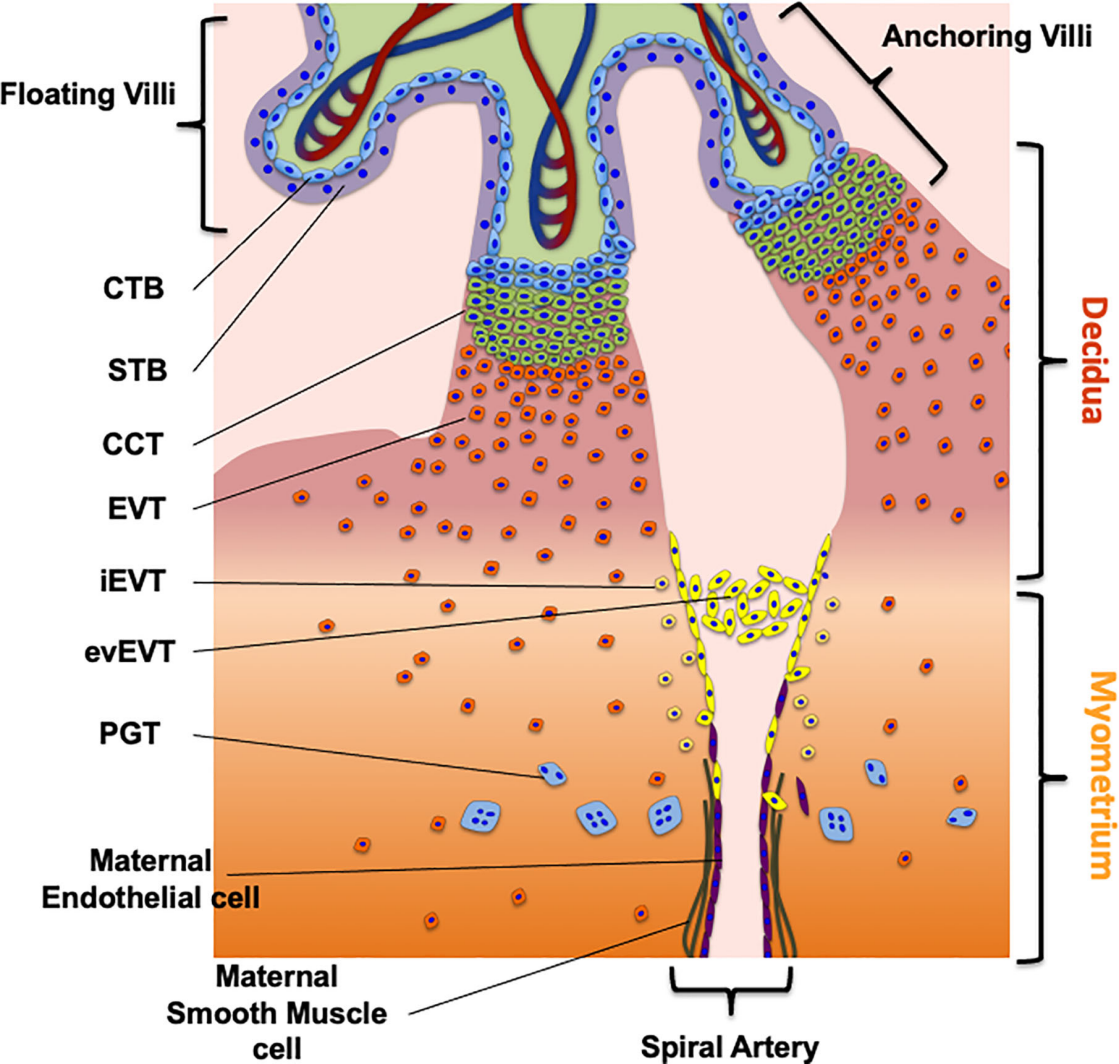
The maternal fetal interface and trophoblast cells subtypes. The figure shows the placental cell types required for the early first trimester human placentation as well as the route to migrate and invade the decidua and myometrium. The different trophoblast subtypes are villous cytotrophoblast (CTB), synctiotrophoblast (STB), cell column trophoblast (CCT), extravillous trophoblast (EVT), endovascular EVT (evEVT), interstitial EVT (iEVT), placental giant trophoblast (PGT). The complete description of the process is in the section “*Development of the Human Placenta*” of the review.

The placenta develops from the trophectoderm (TE), the outer layer of the blastocyst from which derives the undifferentiated cytotrophoblast (CTB). The CTB originates two main villus structures: the floating villus, where CTBs fuse to form the multinuclear syncyiotrophoblast (STB) and the anchoring villus (17–20) (**Figure 1**). The STB acts as an exchange barrier with the maternal blood to assure nutrients as well as waste and gases exchange with the fetal blood (21). The floating villus cells proliferate to form primary villi, which show further branching, forming the intervillous space. The branching to secondary and tertiary villi, allows the expansion of the STB surface area, which favors an efficient nutrient exchange with the fetal blood (19).

As mentioned, the second villus structure derived from the CTB is the anchoring villus, whose main function is to mediate the placental attachment to the endometrium in the uterine wall, to sustain fetal growth (17–20) (**Figure 1**). In the distal tip of the anchoring villus it is possible to find a group of proliferating cells known as cell column trophoblast (CCT). From these cells emerge the placental giant trophoblast (PGT) that mediates the early histotrophic nutrition of the embryo and of extravillous trophoblast cells (EVT). EVTs are a group of non-proliferating trophoblast cells characterized by a highly invasive phenotype. They invade the maternal decidua and the first third of the myometrium, playing a crucial role in the histotrophic nutrition of the fetus, immunomodulation and remodeling of the uterine spiral arteries (**Figure 1**) (19).

These activities are mediated by specialized subgroups of EVTs, characterized by specific markers: the endovascular EVT (evEVT) and the interstitial EVT (iEVT) (**Table 1**). The evEVTs migrate through the lumen of the spiral arteries forming a trophoblast plug reducing the maternal blood flow towards the intervillous space during the early stages of placenta development, permitting histiotrophic nutrition (46). Additionally, evEVT maintain the oxygen concentration low, which is required for placental development and successful trophoblast differentiation and may also promote favorable trophoblast migration and endothelial cell replacement, both required for vascular remodeling. Finally, at the end of first trimester the trophoblast plug formed by evEVT is disintegrated (47, 48).

**Table 1:** Classical markers of trophoblast-derived cells in the human placenta and its expression in vasculogenic mimicry on human cancer.

Type of marker	Marker	CTB	STB	CCT	evEVT	iEVT	Reference	Expression in vasculogenic mimicry on human cancer	Reference
**Epithelial marker**	CK7	+	+	+	NA	+	(22)	–	
**Mesenchymal marker**	Vimentin	–	–	–	NA	–	(23)	Hepatocellular, Colorectal, Ovarian, pancreatic, Large lung cancer, Non-small cell lung cancer, renal cell carcinoma	(24–26)
**Integrins**									
	α1 β1	–	–	–	+	+	(27)	–	
	α5 β1	–	NA	–	NA	+	(27)	Glioblastoma, melanoma	(26, 28, 29)
	α6 β4	+	–	+	NA	–	(27)	–	
	αv β5	+	–	+	–	–	(30)	–	
	αv β3	–	–	–	+	+	(30)	Breast cancer, prostate, colon, melanoma	(31)
**Cell adhesion molecules**									
	VE-Cadherin	–	–	–	+	+	(32)	Melanoma, hepatocellular, Non-small cell lung cancer, colorectal, prostate, large-cell lung cancer, gastric	(26, 33),
	E-cadherin	+	+	+	–	–	(32)	Ovarian, colorectal, pancreatic, large-cell lung cancer, hepatocellular, Non-small cell lung cancer, melanoma	(26, 34),
	PECAM	+	+	+	+	–	(32)	Melanoma	(31)
	NCAM	–	–	–	+	–	(32, 35),	–	
**Metalloproteases**									
	MMP-2	NA	NA	+	NA	+	(36)	Melanoma, ovarian cancer	(34, 37),
	MMP-9	NA	NA	+	NA	+	(36)	Ovarian cancer, Hepatocellular	(26, 34),
	MMP-14 (MT1-MMP)	+	+	+	–	+	(38–40)	Melanoma	(37)
**Hormones**									
	hCG α	+	+	+/-	+/-	+/-	(41)	–	
	hCG β	+/-	+	–	–	–	(41)	–	
	hPL	–	+	+	+	NA	(27)	–	
**Growth factors**									
	TGF β	+	+	+	NA	–	(42)	Hepatocellular	(26)
	VEGF	NA	+	NA	NA	+	(43)	Ovarian cancer	(34)
	sFLT-1	NA	+	NA	NA	+	(43)	–	
	Endoglin	–	+	+	NA	–	(44)	–	
**Immune factors**									
	HLA-G	–	–	–	NA	+	(23, 45),	–	

Classical markers of trophoblast-derived cells in the human placenta and their expression in malign tumors are showed in the table. CK7, cytokeratin 7; VE-cadherin, Vascular endothelial-Cadherin; PECAM, platelet endothelial cell adhesion molecule; NCAM, neural cell adhesion molecule; MMP-2, matrix metallopeptidase 2; MMP-9, matrix metallopeptidase 9; MMP-14, matrix metallopeptidase 14; hCG α, human chorionic gonadotropin α; hCG β, human chorionic gonadotropin β; hPL, human placental lactogen; TGF β, transforming growth factor beta; VEGF, vascular endothelial growth factor; sFLT-1, Fms-like tyrosine kinase-1; EGF, epidermal growth factor; HLA-G, human leukocyte antigen G.

The main function of iEVT is to participate to the immune tolerance and placental invasion of maternal tissues. iEVTs express Human Leukocyte antigen-G (HLA-G), a nonclassical major histocompatibility complex (MHC) (**Table 1**), which is essential to modulate the immune tolerance at the maternal-fetal interface by regulating the interaction and communication with the Uterine Natural Killers (uNK) (49).

iEVTs invade trough the interstitium of the decidua and myometrium towards the maternal spiral arteries (46). Once there, the iEVT acquires an endothelial-like phenotype under conditions of low oxygen concentration (approximately 8% oxygen) replacing the maternal endothelial cells of the uterine spiral arteries. It is important to highlight that many studies refer to this low oxygen concentration as an “hypoxic” environment. However, the reduced oxygen level corresponds to the physiological level required for placenta development, which is maintained until the end of the first trimester (below 20 mmHg). Thus, as this occurs in normal placental development, we will refer to this low oxygen condition as normoxic and not hypoxia (50–52).

The process of invasion by EVTs allows the replacement of the endothelial layer of the maternal spiral arteries, which is essential for an appropriate placental perfusion that maintains an adequate fetal growth. This complex process not only involves different placental cell types, but also a wide range of molecules related to cell growth (i.e., hormones and growth factors), cellular adhesion (i.e., Integrins, Cadherins), tissular remodeling (i.e., Metalloproteases) and proteins related to immune tolerance (**Table 1**) (19, 53). In addition to the classical markers, other molecules including structural and adhesion related proteins, proteins associated to invasion, immunity, embryonic stem cell-associated transcription regulators and oncogenes have been recently described as markers of CTB, STB, iEVT or eEVT (54–58).

CTB and CCT but not EVT cells show proliferative activity and generate cells that stop the proliferation and start to differentiate (20). The process of proliferation and differentiation of CTB into migratory, invasive EVT and endothelial-like trophoblast shows similarities with the process of tumor formation and metastasis of cancer cells. The main similarities are: (i) tissue invasion, (ii) immune system modulation and (iii) vascularization. Despite this, a crucial difference between trophoblast derived and cancer cells is that while in trophoblast cells these processes are regulated, this regulation is lost in cancer cells (4).

The physiological process of placentation responds to a controlled program that results in changes in gene expression and cell cycle. As such, when placentation is not kept under control, malformation of the placenta, pregnancy pathologies and abortions can occur (17, 20, 59). Different abnormal placentation processes have been described, which are characterized by abnormal trophoblast invasion such as abnormally invasive placentas. Abnormal placentation processes are: (i) placenta accreta (abnormal adherence with direct contact to myometrium), (ii) placenta increta (placental villi penetrate into the myometrium) and (iii) percreta (placental villi penetrate trough myometrium to uterine serosa and into the surrounding structures such as the bladder). It has been suggested that trophoblast cells of this abnormally invasive placentas lose their physiological regulation, leading to increased proliferative activity during the invasion, behaving like cancer cells (60, 61).

## Similarities and Differences Between Cancer Cells and Trophoblast Derived Cells

### Cellular Proliferation, Migration, and Invasion

Both cancer cells and trophoblast derived cells express different molecules such as growth factors, proto-oncogenes, enzymes, cell surface receptors, enzyme receptors, hormones and peptides, whose activation mediates their high proliferative, migratory and invasive capacity. During placentation, growth factors such as Epidermal Growth Factor (EGF), Hepatocyte Growth Factor (HGF), Vascular Endothelial Growth Factor (VEGF), Placental Growth Factor (PLGF), Insulin-like Growth Factor (IGF), Transforming Growth Factor (TGF) and their corresponding receptors are among the main factors that regulate CTB proliferation, acting in a paracrine and autocrine manner (62). These growth factors bind to Tyrosine Kinase Receptors to activate the Mitogen-activated Protein/extracellular Signal-regulated kinase/Extracellular Signal-Regulated Kinase (MEK/ERK) proliferation pathway and the Phosphatidylinositol 3-kinase/Protein Kinase B (PI3K/Akt) anti-apoptosis pathway (3). Moreover, proto-oncogenes play an essential role in the etiology of cancer inducing its growth. As cancer cells, trophoblast derived cells express several proto-oncogenes; for example, CTB and STB exclusively express proto-oncogenes that encode Growth Factor Receptor c-erbB1 (Human Epidermal Growth Factor Receptor 1 (HER1), Epidermal Growth Factor Receptor 1 (ERBB1 or EGF-receptor)) (63). Also, trophoblast cells such as CTB, STB, and EVT encode for a Receptor Tyrosine Kinase (RTK), namely c-erbB2 (HER2/neu, ERBB2), c-fms (CSF1R), c-met (MET) and c-kit (KIT) (64–67), as well as for transcription factors that have been implicated in trophoblast invasion such as c-fos (FOS) and c-jun (JUN), in addition to c-myc (MYC) and c-ets1 (ETS) (68–71). Additionally, in iEVT, *c-sis* (SIS, Platelet-derived Growth Factor Beta (PDGFB)) is expressed, which encodes for one of the two chains (the B-chains) constituting Platelet-derived Growth Factor (PDGF) (72) and in EVT the *c-ras* family (Kirsten rat Sarcoma viral oncogene (K-RAS), Neuroblastoma RAS viral oncogene homolog (N-RAS), and Harvey rat sarcoma (H-RAS)) is expressed encoding for Rat sarcoma (RAS) proteins that regulate cellular proliferation and inflammation in the human placenta (73, 74). All the aforementioned proto-oncogenes are crucial in the first step of malignant transformation and its physiological expression occurs during the first week of pregnancy promoting proliferation, migration, and invasion of the trophoblast (2).

The Telomerase is a factor that regulates the proliferative capacity of a cell, as it maintains chromosome stability in actively dividing cells (75). CTB expresses a functional Telomerase, which is downregulated during differentiation, but expressed in term placenta. During human pregnancy, Telomerase activity is the highest during the first trimester, and decreases with the maturation of the placenta (76). Telomerase activity ensures a high rate of proliferation and could be a factor controlling placental growth (77–79). Consistently, in cancer cells, the Telomerase allows uncontrolled cell proliferation, which is essential for tumor progression (80). Additionally, Survivin, a protein overexpressed in many cancers (81), where it promotes proliferation and inhibits apoptosis, is expressed in trophoblast cells, however its role in this location has not been elucidated yet (82, 83). Altogether these studies indicate that the Telomerase and Survivin have an important role in cell proliferation in both trophoblast and cancer cells.

As mentioned, placental development during the first trimester occurs in a stable state of low oxygen concentration (84); by comparison, in tumors, hypoxia is necessary to support tumor growth and metastasis (85). In response to low oxygen levels, cells upregulate Hypoxia-Inducible Factor (HIF), a family of transcription factors that functions as a Heterodimer with a regulatory α subunit (HIF-α) and a constitutive β subunit (HIF- β) (86, 87). The activation of the different HIF isoforms leads to the transcription of genes involved in several processes such as metabolism, angiogenesis, and immunomodulation (86). Thus, this low oxygen concentration environment in trophoblast and cancer cells could be considered as key to stimulate proliferation, invasion, and vasculogenesis in host tissues (88).

During placentation and cancer growth, invasion is required to provide blood and nutrient supply. Different events need to occur for a successful invasion process: (i) changes in the expression of Cell Adhesion molecules (ii) secretion of Proteases, and (iii) availability of Growth Factors (5). One feature shared by both cell types is the process of epithelial to mesenchymal transition (EMT), which leads to the loss of cell-to-cell contact inhibition, and to the increased expression of proteins that degrade the extracellular matrix. During EMT the Integrin expression pattern changes, and the expression of E-cadherin decreases, enhancing cell movement through tissues by reducing cell polarity (89, 90).

EVT and invasive cancer cells also share enzymes required for the degradation of the basal membrane that allow the process of invasion. Among those there are Serine Proteases, Cathepsins and Matrix Metalloproteinases (MMPs), the Heparan Sulfate-degrading Endoglycosidase, the Protease-Activated Receptor (PAR) and the Receptor of Thrombin (91, 92). These enzymes are expressed transiently in the trophoblast, in a very regulated manner, while in cancer cells their expression becomes constitutive (2, 4). As an example, the expression of MMP-2 and MMP-9 is increased during trophoblast invasion, promoting proteolysis and therefore invasion. Importantly, when the invasion is completed, decidual cells inhibit MMP-2 and MMP-9 activity by the release of protease inhibitors (53, 93, 94). When the control of the protease activity is lost abnormally invasive placentas develop, consistently, this regulation disappears in cancer invasive cells (60).

Additionally, Placenta-Specific Protein 8 (PLAC-8) is a placental protein implicated in embryo implantation, which is expressed in iEVT on the feto-maternal interface promoting trophoblast invasion and migration (57, 95) nevertheless PLAC-8 is also expressed in cells from different cancers such as lung adenocarcinoma, pancreatic cancer, colorectal cancer, gastrointestinal cancer, and cervical cancer (96–100), where it is involved in malignant tumor progression by regulating cell differentiation (100), proliferation (101), apoptosis (102) and autophagy by mediating autophagosome/autolysosome fusion (103).

In conclusion, for the physiological invasion of iEVT and for the pathological metastasis of cancer cells similar mechanisms are used. However, despite the similarities between them, they show a key difference: while the trophoblast follows an organized pattern of proliferation, differentiation and invasion without metastasizing to new tissues; cancer cells spread through the host tissue with a high proliferation rate, with the final objective of being able to metastasize to other tissues (3, 5).

### Vasculogenic Capacity

The vascularization capacity is also a common feature between trophoblast and cancer cells as an abundant blood supply is necessary both for the growth of the tumor nodule, and for the implanting embryo. To date, three processes of vessel growth have been described: vasculogenesis, angiogenesis and vascular mimicry (104, 105). Vasculogenesis is the process of new blood vessel formation from angioblast precursor cells; angiogenesis is the process of growth and development of new capillary blood vessels from pre-existing vessels like new branches; vascular mimicry corresponds to vessel growth from adult cells into a vascular-like phenotype (105, 106).

During the first trimester of pregnancy, vasculogenesis and angiogenesis are consecutive processes. Mesenchymal stem cells differentiate to become hemangiogenic stem cells, then, in a paracrine manner, the CTB induces the formation of the first vessels *via* induction of VEGF signaling. After that, the existing vessels become longer, a process mediated by VEGF and PLGF (107). In cancer, angiogenesis is crucial for the newly formed tumor nodule, since it provides blood continuously to initiate progression and tumor growth (108). This process involves molecular and cellular interactions between cancerous cells, endothelial cells, and some components of the Extra-Cellular Matrix (ECM), such as matrix proteins (Fibronectin, Laminin, Collagen), receptors (Integrins) and enzymes that degrade the ECM [MMP and Tissue Inhibitor of Metalloproteinase (TIMP)]. Specific proteins such as VEGF and FGF are secreted by cancer cells to stimulate the proliferation of capillary endothelial cells leading to the sprout and branching of them through the ECM (109). Recent evidence suggests that in tumors resistant to different anti-angiogenic drugs, in addition to angiogenesis, other processes that contribute to tumoral vascularization occur, namely vasculogenesis and vascular mimicry (110, 111).

Interestingly, human EVT and invasive cancer cells have similar patterns of integrins expression (**Table 1**), which allows the EVT to adopt a vascular phenotype capable of invading maternal spiral arterioles, a process similar to what occurs in endothelial cells when they migrate towards the tumor (5). This turnover of endothelial cells to form new vessels requires different angiogenesis regulators that are similar between EVT and cancer cells (3). Among those, VEGF and PLGF promote angiogenesis and are regulated by hypoxia and Fibroblast Growth Factor (FGF) can initiate angiogenesis in both cell types. Conversely, Angiostatin, Fibronectin, and Tissue inhibitor of Metalloproteinases act as angiogenesis inhibitors (109, 112, 113).

Additionally, both cell types are able to directly contribute to their own blood supply by inducing vascular mimicry (88), enhancing gene expression patterns and signaling pathways shared by the two cell types (5). As an example, the Galactose-binding protein Galectin-3, which is known to provide a vascular phenotype, is highly expressed in EVT (114) and is also a key factor for the development of aggressive melanomas (115).

In summary, the process of angiogenesis is essential both in trophoblast and cancer cells. In cancer it drives tumor growth and metastasis, and in pregnancy it allows proper embryo implantation and placentation. However, while trophoblast cells create new blood vessels inducing a controlled process of vasculogenesis, the angiogenesis in cancer is uncontrolled (3).

### Immune Evasion

For proper development, trophoblast and cancer cells evade the immune response of the host. During placentation, for the development of the maternal-fetal interface, the maternal decidua basalis, where the maternal immune cells are located, interacts with the fetal derived placental iEVT. Additionally the placenta produces anti-inflammatory Cytokines, TGF-β2, Interleukin (IL)-4 and IL-10, which reduce the deleterious effects of pro-inflammatory cytokines (4). Fas Ligand (Fas-L) expression on trophoblast promotes apoptosis of Fas-expressing lymphocytes of maternal origin, having a role in placental invasion during implantation (116). The position of trophoblast cells in the placenta encasing the embryo produces a barrier between maternal and fetal cells, finally being the placenta the main separation of fetal and maternal blood and lymphatic systems, preventing the immune system of the mother to perceive fetal antigens. During the first trimester the immune cells located in the decidua basalis are Natural Killer (NK, 70%), Macrophages (20-25%) and T Lymphocytes [3-10%, (117–119)]. It has been suggested that the presence of progesterone and TGF-β1 in the decidua promotes the differentiation of these NK into mature Uterine Natural Killers (uNK) (120, 121). uNK cells are more immunomodulatory than cytotoxic, they secrete Growth factors, Angiogenic factors and Cytokines facilitating immune tolerance which suggests uNK play a role in implantation, invasion and vascular remodeling of spiral artery remodeling, regulating EVT invasion (122) by a mechanism that has not yet been totally clarified (49, 123, 124). Additionally, macrophages have been shown as capable of regulating the process of spiral artery remodeling, metabolic regulation of lipids, tissue regeneration, inflammation and fetal antigen recognition (125). Furthermore, they can influence EVT function as they are more abundant at the invasive front and implantation site (126, 127). Despite these studies, their role in placentation, as support of trophoblast cells, has not been fully elucidated. The role of T lymphocytes is also poorly understood, however, it has been described that they could have a role in controlling infections caused by bacteria located at the maternal-fetal interface (119).

In cancer, NK cells are known to contribute to tumor development *via* secretion of Cytokines (128, 129). Additionally, cancer cells express tumor-associated Macrophages, which can have an inflammatory and immunosuppressive role, being key in tumor progression and metastasis (130). Fas-mediated apoptosis and the expression of Fas-L allow many cancers to attack the immune system (131, 132). Regulatory T cells are implicated in mediating tolerance in cancer and pregnancy; immunophenotypically expressing Cluster of differentiation (CD); CD4, CD25 and Forkead Box P3 (FOXP3) (133). In pregnancy, regulatory T cells are induced by paternal/fetal alloantigens (134), which is crucial for maternal-fetal tolerance. In cancer, regulatory T cells are implicated in impaired antitumor immunity, suppression of effector T lymphocytes proliferation, and increased tumor blood vessel density, suggesting an essential link between immunity and angiogenesis (5). iEVT express Human leukocyte antigen- G (HLA-G) (19), which suppresses cytolytic killing by NK and cytotoxic T cells inducing apoptosis of immune cells (49). HLA-G regulates cytokine production in blood mononuclear cells, reducing stimulatory capacity and impairing the maturation of dendritic cells (5). In tumors, HLA-G promotes immune evasion by interacting with NK cells *via* Inhibitory receptors and Killer cell Immunoglobulin-like receptor (KIR) (135). This molecule can directly mediate immune tolerance by inhibiting receptors, predominantly Immunoglobulin-like Transcript (ILT) 2 and 4 expressed on immune effectors (136). Finally, HLA-G has been detected in melanoma and solid tumors including cervical cancer, gastrointestinal cancer and breast cancer (137–139).

In conclusion, both trophoblast and cancer cells actively modulate the host immune response by different mechanisms that are induced by similar cells and molecules, finally promoting cell invasion.

## Autophagy

**Figure 2:**
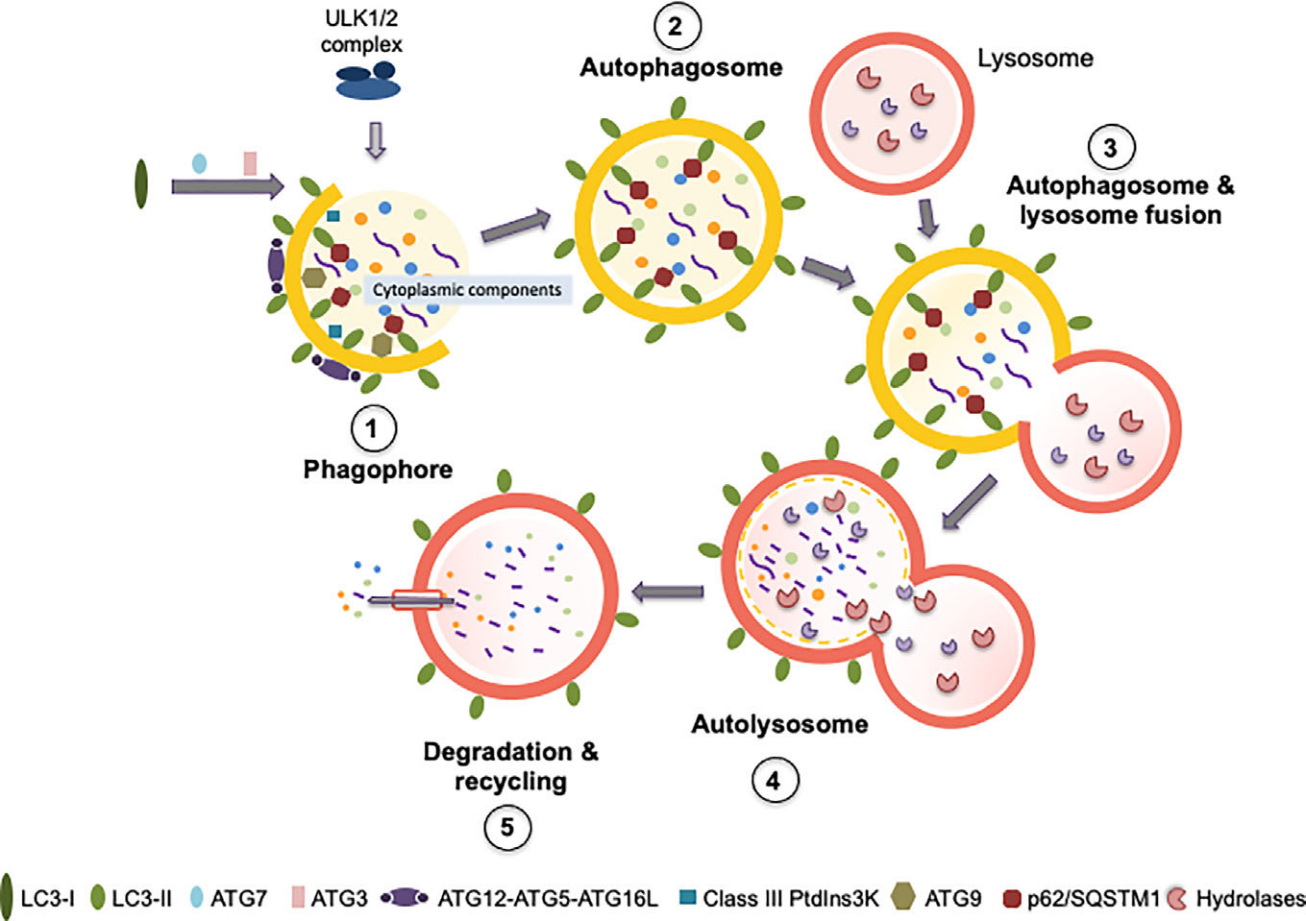
Autophagy process and principal proteins involved in the different steps. The figure shows the principal proteins required for autophagy process, the different steps of the process are described in the section “*Autophagy*” (initiation, nucleation, elongation, fusion with the lysosome, and cargo degradation and recycling). Figure (1) corresponds to phagophore formation that includes initiation and nucleation. (2) Autophagosome maturation includes the elongation process. (3) Autophagosome and lysosome fusion. (4) Represent the structure of the autolysosome, and (5) corresponds to degradation and recycling. In each step are indicated the main proteins required: ULK1/2 complex, LC3 I, LC3II, ATG7, ATG3, ATG12-ATG5-ATG16L, Class III Ptdlns3K, ATG9 and p62/SQSTM1. The yellow semi-circumferences and circumferences correspond to the phagosome membrane. See the main text for further details.

Autophagy is a catabolic process highly conserved among eukaryotic organisms, which allows the lysosomal-mediated degradation of cytoplasmic components, thus contributing to cell homeostasis. Three types of autophagy have been described based on the mechanism by which the cargo is delivered to the lysosome: (i) microautophagy, where the cytosolic material is delivered to the lysosome by a direct invagination or protrusion of the lysosomal membrane (140) (ii) chaperone-mediated autophagy, where unfolded soluble proteins containing a specific consensus motif translocate across the lysosomal membrane (141–143), and macroautophagy, herein referred to as autophagy, where the cargo is sequestered in a special double membrane organelle known as autophagosome and then delivered to the lysosome. Briefly, during autophagy the autophagosome fuses to lysosome, forming the autolysosome, where the cargo is degraded (144) (**Figure 2**). The new metabolites derived from the degradation return then back to the cytosol and will be used for the synthesis of new macromolecules and/or energy production (145). Different autophagy-related (ATG) proteins are required for autophagy to occur, these are organized in protein complexes that are necessary in the different steps of the autophagic process. These can be divided into five stages (initiation, nucleation, elongation, fusion with the lysosome, and cargo degradation) (**Figure 2**). During “initiation” the unc-51-like kinase 1 (ULK1)/focal adhesion kinase family interacting protein of 200 kDa (FIP200)/ATG13 complex (ULK1 complex) is activated, in response to the metabolic status of the cell (146). Once active, the ULK1 complex translocates to membranous sites, known as omegasomes, where the autophagosome will form (i.e. endoplasmic reticulum and mitochondria contact sites) (147, 148). Then, during the “nucleation”, the isolation membrane of the new autophagosome is generated. This process is mediated by the kinase complex formed by Vacuolar Sorting Protein (VPS) 34 (VPS34), Beclin-1, and VPS15 and Autophagy related 14-like protein (ATGL14), which generates phosphatidylinositol 3-phosphate (PI3P), necessary for the recruitment of the machinery required for the generation of the new autophagosome (146, 149). ATG9-containing vesicles cycle between the omegasome and the Golgi/endosomes, and they contribute to the recruitment of membranes for the nucleation of the phagophore (147, 150, 151). Then, the phagophore extends during the “elongation stage”, a process that is tightly regulated by two ubiquitin-like systems: the microtubule-associated protein 1A/1B-light chain 3 (MAP1LC3A, also known as LC3-I) system and the ATG5–ATG12 system (152) and by the ATG5–ATG12 complex. The ATG5–ATG12 complex then interacts with ATG16L, forming a new complex that works like an E3 enzyme, assisting the incorporation of LC3-II into the membrane of the phagophore (153). In parallel with the elongation the autophagic cargo is selected. Proteins targeted for autophagy are labeled with the receptor p62/Sequestosome-1 (p62/SQSTM1), which interacts with LC3 through an LC3 interacting region (LIR) (154, 155). Following elongation, the elongated phagophore is finally closed forming the autophagosome. This step is completed by a membrane abscission process mediated by the endosomal-sorting complex required for transport (ESCRT) (156, 157). Upon closure, the nascent autophagosome dissociates from the assembly site and undergoes maturation (158). The mature autophagosome then fuses with the lysosome generating autolysosomes (159), a process mediated by Rab GTPases, membrane-tethering complexes and soluble N-ethylmaleimide-sensitive factor attachment protein receptors (SNAREs) (160). The inner membrane of the autolysosome breaks down and the process of autophagosomal cargo degradation begins (161). The degradation products are recycled and turn back to the cytosol for being reused (162, 163) (**Figure 2**).

Autophagy is a complex and highly regulated process that under stress conditions such as hypoxia, low glucose concentration and oxidative stress is triggered to promote cell survival or leads to cell death. Physiologically autophagy maintains cellular and energy homeostasis, cooperates with the immune system to promote adaptation, and represents a quality control system for proteins and organelles (164). Impaired autophagy contributes to the development of neurodegenerative (165), infectious (166) and metabolic diseases (167, 168), due to the accumulation of abnormal and damaged proteins and/or organelles, forming intracellular aggregates that induce cellular stress, finally promoting cell death.

During placentation it has been reported that autophagy could be relevant for different processes required for a proper development of the placenta; however, how this occurs is still under investigation (7). On the other hand, in the context of cancer, autophagy has a dual role, where it could be tumor-suppressive or tumor-promoting depending on the stage of cancer development and the type of cancer considered (10).

## Role of Autophagy in Immune Evasion and Vascular Remodeling: Differences Between Placentation and Cancer

As previously described, the processes of placentation and tumor development share similarities and autophagy activation has been described in both (6, 7). The role of autophagy in cancer has been widely explored, however, as previously mentioned, its role has not completely been elucidated (169). On the other hand, how modulation of autophagy affects trophoblast function is still largely unknown.

### Role of Autophagy in Placental and Cancer Immune Evasion

The role of autophagy in the placentation process remains unclear, and its contribution to immune evasion is still unknown (170). It has been described that autophagy is highly activated in decidualized endometrium of early pregnancy, which increases NK cell adhesion and retention in the decidua. Also, when autophagy is inhibited, decidual NK (dNK) cell residence is decreased, contributing to spontaneous abortion (171). Tan et al. described that autophagy levels are highly reduced in cases of recurrent miscarriage. Indeed, suppression of autophagy in an *in vitro* model of trophoblast cells enhances the cytotoxicity activity of dNK, impairing trophoblast invasion, finally causing abortion (172).

On the other hand, autophagy has been describes as an important regulator of cancer immunity in the tumor microenvironment; however, the exact mechanisms involved remain unclear (173, 174). The tumor microenvironment contains different factors that promote autophagy, such as hypoxia or inflammation (166, 175). Remarkably, it has been described that autophagic activation correlates with immune evasion (176, 177). Conversely, inhibition of autophagy associates with NK-dependent immune responses. In breast cancers, in tumors presenting hypoxia, blocking autophagy restores NK-mediated lysis *in vitro*, facilitating breast tumor elimination by NK cells in mice (178). Inhibition of autophagy also reduces NK cell-mediated cytotoxicity in melanoma (175), non-small cell lung cancer (179) and liver cancer (180). In contrast, the role of autophagy is dual in the response to immune cell recognition, being a suppressor or inductor of tumorigenesis depending on the specific context (181). Altogether these data suggest that autophagy actively participates and regulates the immune evasion of dNK in placental development and NK activity in cancer cells. However, the mechanism involved in both phenomena remains to be elucidated, a crucial aspect that needs to be studied for the development of immunotherapy in each field.

### Role of Autophagy in Placental Vascular Remodeling

As indicated, trophoblast invasion and vascular remodeling allows the replacement of the endothelial layer of the maternal spiral arteries, which is essential for proper placental perfusion and adequate fetal growth. It has been described that activation of autophagy occurs in human placentas from normal pregnancies at weeks 8 to 12 of gestation, as indicated by LC3 and Beclin-1 protein in CTB and STB cells (182) (**Table 2**). Moreover, autophagosomes have been identified in human placentas throughout gestation from early (8 weeks) (189, 194) to term pregnancies (39 weeks) (189, 194, 261).

**Table 2:** Changes in protein involved in the autophagic process described in human placental tissues and trophoblast cell lines.

Study model			
Human placental tissue	Cell line	Autophagy marker	Reference
CS vs VD	–	↑LC3	(183)
PES vs N	–	↑LC3, ↑Beclin-1	(184)
IUGR vs N	–	↑LC3, ↑Beclin-1	(185)
–	HTR-8/SVneo inhibition of ﻿Hypoxia inducible factor (HIF)-1α	↑LC3, ↓Beclin-1	(186)
CTB exposed to hypoxia vs normoxia	–	↑LC3, ↑p62	(187)
MC sIUGR vs MC	–	↑LC3	(188)
EVT exposed to hypoxia vs normoxia	HTR-8/SVneo exposed to hypoxia vs normoxia	↑LC3, ↓p62	(189)
FTP, N	–	LC3, Beclin-1	(182)
SP vs IL	–	↑LC3	(190)
NE vs N	–	↑LC3	(191)
–	HTR-8/SVneo exposed to Cobalt chloride (CoCl_2_)	↑LC3	(192)
PE vs N	JEG-3	↑LC3	(193)
FTP,MD, N, CS, VD	–	= LC3, = Beclin-1	(194)
N		↑LC3, ↑ATG5-12	(195)
	JEG-3	↑LC3	
PIH vs N	–	↑LC3, ↓p62	(196)
PE vs N HUVEC	HTR-8/SVneo	↑LC3, ↑Beclin-1	(197)
Early placenta-SM vs Normal-early placenta	–	↑LC3	(198)
–	JEG-3 with ﻿ASAH1 inhibition or ceramide treatment	↑LC3, ↑p62	(199)
Fetal membrane SP vs Fetal membrane N	–	↓Beclin-1, ↓ ATG3, ↓ATG5, ↓ATG7, ↓ATG12, ↓AT16L1	(200)
–	BeWo ﻿treated with dexamethasone	↑LC3	(201)
PE vs N	–	↑p62	(202)
STB ﻿treated with punicalagin	–	↓ LC3, ↓p62	(203)
OB vs N	–	↑Beclin-1, ↑ATG3, ↑ATG7, ↑LC3	(204)
–	BeWo exposed to an increase in reactive oxygen species	↑ATG5, ↑ATG7, ↑LC3, ↓p62	(205)
PE vs N	JEG-3	↓LC3, ↓Beclin-1	(206)
PTD vs N		↓LC3, ↑p62, ↓ ATG6L, = Beclin-1, = ATG7	(207)
	BeWo	↑LC3	
PES vs N	HTR-8/SVneo, JEG-3	↑LC3, ↑ATG4B	(208)
PTD with/without inflammatory lesions	–	↑LC3	(209)
EOPE vs N	–	↑LC3	(210)
GDM vs N	–	↓Beclin-1, ↑LC3, ↑p62	(211)
FGR vs N	–	↑LC3, ↑ Beclin-1, ↓ p62	(212)
GDM vs N		↑LC3, ↓ p62	(213)
	HTR-8/SVneo	↑LC3, ↓ p62, ↑ATG5	
FTP vs N	–	↓LC3	(214)
IUGR, EOPE vs N	–	↑LC3, ↑ Beclin-1	(215)
Placenta with Malaria vs N	–	↑LC3, = ATG4B, = p62	(216)
–	BeWo ﻿exposed to overexpression of CYP11A1 gene	↑LC3, ↑ Beclin-1	(217)
IUGR vs N	–	↑LC3, ↓ p62	(218)
PE vs N	–	↑Beclin-1, ↑p62	(219)
–	JEG-3 exposed to cigarette smoke	↑LC3, ↑p62	(220)
FTP Primary trophoblast	BeWo	↑LC3, ↑p62	(221)
–	Sw.71 exposed to saturated fatty acids	↑p62, ↑LC3	(222)
–	BeWo exposed to cobalt and chromium nanoparticles	↑LC3, ↑p62	(223)
PE vs N	–	↑LC3, ↑p62	(224)
PES vs N	–	↑LC3, ↓ p62	(225)
CTB	–	↑LC3, ↑ Beclin-1	(226)
ICP vs N	HTR-8/SVneo	↑LC3, ↑ATG5, ↑ ATG7, ↑Beclin-1	(227)
–	HTR-8/SVneo exposed to ﻿Titanium dioxide nanoparticles	↑LC3, ↑p62	(228)
Early miscarriage vs N	–	↑ LC3, ↑ATG5, ↑Beclin-1	(229)
–	HTR-8/SVneo exposed to ﻿Titanium dioxide nanoparticles	↑LC3, ↑p62	(230)
–	HTR-8/SVneo, JEG-3 associated to long noncoding RNA H19 downregulation	↑LC3, ↑Beclin-1, ↓ p62	(231)
CTB	BeWo	↑ATG16, ↑ATG5-ATG12, ↑ATG7, ↑LC3, ↓p62	(232)
–	HchEpC1b, HTR-8/SVneo exposed to ﻿platinum nanoparticles (npt)	↑LC3, ↓ p62	(233)
HDCP vs N	HPVEC	↓Beclin-1,↓LC3	(234)
Placenta accrete vs N	–	↑LC3, ↑Beclin-1, ↑p62	(235)
–	HchEpC1b exposed to ﻿oxidative stress	↑p62,	(236)
–	JEG-3 exposed to Cadmium	↑LC3, ↑p62	(237)
CTB	BeWo	↑LC3	(238)
Plasmodium falciparum-infected women vs non infected	–	↓Beclin-1,↓LC3	(239)
–	HTR-8/SVneo ﻿with inhibition of Death-associated protein kinase-3	↑LC3, ↑p62,↑ATG5	(240)
–	HTR-8/SVneo exposed to hypoxia	↑LC3	(241)
–	HTR-8/SVneo exposed to ﻿oxidative stress	↓Beclin-1,↓LC3, ↑p62	(242)
–	HTR-8/SVneo with knockdown of plasmacytoma variant translocation 1	↓Beclin-1,↓LC3, ↑p62	(243)
–	HTR-8/SVneo exposed to Hydrogen peroxide	↑LC3, ↑Beclin-1	(244)
GDM vs N CTB	–	↓Beclin-1, ↓ATG5, ↓LC3, ↓p62	(245)
Anemic vs polycythemic territories in TAPS	–	↑LC3, ↑p62	(246)
–	BeWo ﻿under hyperglycemic conditions reduce	↓LC3, ↓p62	(247)
﻿With vs without Probiotic supplementation in SP	–	↓Beclin-1	(248)
OB vs N	–	↓LC3	(249)
–	HTR-8/SVneo with overexpression of homeobox protein A7	↑LC3, ↓p62	(250)
PE vs N	–	↑LC3	(251)
–	HTR-8/SVneo exposed to high glucose	↑LC3, ↓p62	(252)
GDM vs N	–	↑LC3, ↑ATG7	(253)
DMSC from EPSM vs DSC normal pregnancy	–	↑P62, ↓ LC3, ↓ATG5	(171)
–	JAr exposed to ﻿cyclopamine and/or Gant61	↑LC3	(254)
With vs without mycophenolic Acid treatment in DMSC normal pregnancy	–	↑LC3, ↓p62	(255)
FGR vs N	BeWo	↑LC3, ↑Beclin-1	(256)
N exposed to hypoxia	BeWo exposed to hypoxia	↑LC3, ↑p62	(257)
–	HTR-8/SVneo ﻿exposed to α-solanine	↑LC3, ↑Beclin-1↑ATG13, = p62	(258)
PE vs N	–	↑p62, = LC3	(259)
–	HTR-8/SVneo and Jar with Placenta specific 8 (PLAC8) overexpression	↑ATG5-ATG12, ↑Beclin-1, ↓LC3	(260)

Changes in protein involved in the autophagic process described in human placental tissues and trophoblast cell lines are showed in the table. Human placental tissue abbreviations: N, normal term pregnancy; FTP, first trimester placenta; MD, midgestation; CS, cesarean section; VD, vaginal delivery; PE, preeclampsia; PES, severe preeclampsia; IUGR, intrauterine growth restriction; FGR, fetal growth restriction; CTB, cell primary culture from human placenta cytotrophoblasts; STB, cell primary culture from human placenta syncytiotrophoblasts; MC, monochorionic twin; MC sIUGR, monochorionic twin with selective intrauterine growth restriction; EVT, cell primary culture first trimester extravillous trophoblast; SP, spontaneous labor; IL, induced labor; NE, neonatal encephalopathy; EOPE, early-onset preeclampsia; HUVEC, human umbilical vein endothelial cells; PIH, pregnancy-induced hypertension; EPSM, spontaneous miscarriage; OB, maternal obesity; PTD, preterm delivery; GDM, gestational diabetes mellitus; HDCP, hypertensive disorder complicating pregnancy; ICP, intrahepatic cholestasis of pregnancy; TAPS, monochorionic twin anemia-polycythemia sequence; DMSC, decidua mesenchymal stromal cells from human placenta. Cell line abbreviations: HTR-8/SVneo, human first-trimester extravillous trophoblast cell line; JEG-3, human choriocarcinoma JEG-3 cell line; BeWo, human placental choriocarcinoma cell line; Sw.71, human first trimester trophoblast cell line; HPVEC, human placental microvascular endothelial cells; HchEpC1b, extravillous trophoblast cell line; JAr, human choriocarcinoma trophoblast cell line.

The key role of autophagy in implantation was demonstrated by studies in ATG5-deficient mouse oocytes, where pre-implantation cannot occur correctly. Indeed, autophagy increases in the oocytes after fertilization, and it is necessary for pre-implantation development, which is essential to allow the differentiation from zygote to blastocyst in mammals (262). In different mouse models it has been shown that proteins of the LC3 family are expressed in the labyrinth zone and in the decidua basalis, which suggests a possible role in the placentation process (263).

*In vitro* assays in the trophoblast cell line HTR8/SVneo (i.e., a first-trimester human trophoblast cell line) showed increased LC3 lipidation and LC3 puncta in cells cultured in 2% of oxygen, which mimics the physiological O_2_ concentration in the early pregnancy period (186, 189, 192). In the same cell line, higher LC3 and Beclin-1 expression was determined in conditions of enhanced oxidative stress (264). Additionally, using a model of autophagy-deficient EVT cells (cells expressing a ATG4B- negative mutant), the relevance of autophagy in the trophoblast in the process of invasion was shown, as the process was impaired in autophagy-deficient cells (189). Consistently, in a mouse model where the ATG7 gene was deleted only in trophoblast (not in fetuses), the placentas were smaller than in wild type, due to reduced trophoblast invasion and low vascular remodeling. Remarkably, this result needs to be compared with those described in cancer cells lacking ATG7, which is described in the next section. Altogether, these studies demonstrate that autophagy plays a key role in trophoblast function, especially in invasion and vascular remodeling during placentation (**Table 2**). Despite this, how modulation of autophagy affects trophoblast function in pathological conditions has not been elucidated.

Importantly, even if previous research indicates a positive correlation between autophagy and cell invasion and vice-versa (192, 264), the role of the whole autophagic process, intended as autophagic flux, defined as the whole process from autophagosome formation up to its fusion with lysosome and cargo degradation (7), in the development of pregnancy-associated diseases such as preeclampsia (PE), gestational diabetes, or fetal growth (FGR) is still controversial. Indeed, for example, in homogenized tissue from PE placenta and in trophoblast cells obtained from PE placentas has been described that LC3 and Beclin-1 are increased (197, 215), suggesting that increased markers of autophagy correlate with a poor placentation process (187). However, another study in PE placenta and in the cell line JEG-3 showed an increase in LC3 without changes in Beclin-1 (265). Furthermore, additional work showed, in homogenized tissue from PE placentas, a decrease in LC3 and Beclin-1 (206) and an increase in Beclin-1 and p62/SQSTM1 (219). These controversial results could be due to different factors: (i) the placenta is a complex organ, with different cell types that perform different functions, so it is not appropriate to use placenta homogenates and evaluate autophagy in these samples, as the levels of autophagy can be different in the different cell types. (ii) The time at which the analysis is performed is important. Indeed, as reported, it has been described that autophagy plays different roles in embryogenesis and implantation, while its role in the later stages of pregnancy is still unknown (8). (iii) It is key to evaluate a set of autophagic markers to study the autophagic flux to reach a conclusion (at least LC3 and an autophagic receptor such as p62/SQSTM1), unfortunately, some of the studies only evaluate a single autophagic protein, which is not sufficient to clearly indicate what is happening in autophagy but only suggest that the condition reported might affect this cellular process (**Table 2**). Thus, the available information related with the role of autophagy in placentation in terms of specific cells involved, cellular processes affected beyond migration of invasion (i.e., processes of differentiation to endothelial phenotype, angiogenesis, vasculogenesis or immune control) and the modulation of autophagy according to gestational age, as well as the complete autophagic flux in these different processes still needs to be elucidated.

### Role of Autophagy in Vascular Remodeling in Cancer

As mentioned, the role of autophagy in tumor development is controversial and dependent of the tumor characteristics and stage of tumor development (266). Briefly, it has been suggested that autophagy could promote aggressive characteristics of cancer cells such as increased cellular invasion (11, 12), but it also represents a barrier for cancer proliferation (13–15).

In cancer cells, the inhibition of autophagy results in impaired metabolism proliferation, survival, and spontaneous tumor malignancy depending not only on the tumor type but also of its temporal development (267). This has been demonstrated in different types of cancers using genetically engineered mouse models with ablation of ATGs and consequently autophagy. For instance, in pancreatic ductal adenocarcinoma, loss of ATG5 increases tumor initiation but avoids invasive cancer progression (268). Consistently, in prostate cancer, lack of ATG7 delayed tumor cell proliferation (269) and in lung cancer driven by oncogenic Kras, the deletion of ATG7 reduces cell proliferation and tumor weight compared with mice with intact ATG7 (270). Conversely, Rao et al. show that ATG5 deletion accelerates early oncogenesis, increasing the number of tumor foci and the transition from hyperplasia to adenomas; however as cancer develops, lack of ATG5 reduces the progression from adenoma to adenocarcinoma, resulting in a decrease of tumors mass and enhanced lifespan in mice (271). Altogether, these studies demonstrate that autophagy plays a crucial role in cancer cells.

According to the stage, during the early phases of solid tumor formation, autophagy plays an anti-tumorigenic (272) effect because it limits the production of DNA damaging agents [i.e. Reactive Oxygen Species (ROS)], it promotes the elimination of oncogenic proteins, and stimulates the induction of the immune response in response to cellular stress (273). Additionally, it has been shown that autophagy could promote senescence in tumor cells in response to oncogenic stress, which results in decreased tumor growth (274, 275). On the other hand, it has been described that during tumor progression, autophagy increases the tolerance to stressful conditions such as metabolic changes and hypoxia within the tumor microenvironment, leading to enhanced tumor cell survival and playing a pro-tumorigenic role (276, 277). Autophagy can also increase metastasis, supporting tumor growth, interacting with pathways involved in cell motility and invasion (6), including the promotion of Focal Adhesion (FA) turnover, which is a component of the cell migration machinery, being Paxillin the essential FA protein degraded by autophagy (278) and ECM proteins. For example, in pancreatic ductal adenocarcinomas hypoxia induces autophagy resulting in degradation of Lumican, an extracellular matrix protein highly upregulated in different cancers (279). Autophagy is also enhanced upon oncogenic RAS activation (280, 281) and is required for the production of multiple secreted factors, which include IL-6 and MMP2 in tumors bearing RAS mutations, facilitating cancer cell invasion (282). All the described data indicate that cancer cell migration could be molecularly regulated by autophagy and vice versa, providing metabolites and nutrients in stress conditions to the different cell types that form the tumor microenvironment (169).

Thus, autophagy has a dual role in cancer since in tumor initiation limits DNA damage agents such as ROS and increases tumor cell senescence leading to an anti-tumorigenic environment, preventing tumor promotion. However, in established tumors autophagy provides the necessary conditions for tumors to growth, regulating the invasion and migration process enhancing tumor cell survival increasing resistance to stressful conditions (176). Something similar occurs in trophoblast cells, where it has been suggested that autophagy regulates invasion, migration and vascular remodeling of trophoblasts, allowing the optimal development of the placenta (7). One difference between both processes is that autophagy has a role in the promotion of the placentation process from fertilization, whereas, as mentioned above, at the beginning of tumor development autophagy exerts anticarcinogenic functions protecting the host tissue, but as the tumor progresses, autophagy supports tumor metastasis, enhancing tumor cell survival by increasing the resistance to stressful conditions (283). Finally, the role of autophagy in cancer cells and trophoblast derived cells appears quite similar, since it provides the conditions to carry out cellular functions depending on the timing or stage in cancer, promoting or stopping tumor growth, while in the trophoblast autophagy could favor optimal placentation. Nevertheless, the precise role of autophagy in modulating the described cellular processes involved in vascular remodeling in cancer progression or placentation needs to be fully studied.

## Conclusion

In conclusion, the physiological placentation process of trophoblast and the pathological metastasis of cancer cells share similar mechanisms to proliferate, migrate, and invade both trophoblast and cancer cells, modulating host immune response. However, the main difference is that trophoblast follows an organized pattern without metastasizing new tissues. On the other hand, another shared process is autophagy, which is required for invasion of trophoblast, and it has been shown in cancer has a dual role being a tumor promoter and inhibitor, depending on the stage and tumor considered. Nevertheless, the precise role of autophagy in cancer progression or placentation needs to be thoroughly studied. These studies could give a new insight in cancer biology by evaluating the similarities with trophoblast cells and the highly regulated behavior they have in placentation.

## Author Contributions

LC and JG: review of the literature. AL and EM: preparation of the manuscript. All authors contributed to the article and approved the submitted version.

## Funding

This work was supported by Fondo Nacional de Desarrollo Científico y Tecnológico (FONDECYT 1180935 JG, 1200499 EM, 1190250 AL). LC holds ANID (Chile) fellowship.

## Conflict of Interest

The authors declare that the research was conducted in the absence of any commercial or financial relationships that could be construed as a potential conflict of interest.
